# Health Tract, No. 37

**Published:** 1865

**Authors:** 


### HEALTH TRACT, No. 37.

### PRECAUTIONS.

1. Never sleep in a room where there is any green paper on the walls, as this color is made of arsenic or lead; the former is by far the most dangerous, being scheeles green, and is known positively by a drop of muriatic acid on the green leaving it white.

2. White glazed visiting-cards contain sugar of lead, and will poison a child who is tempted to chew them from the slight sweetish taste.

3. Green glazed cards used for concert-tickets, are still more poisonous; a single one of them contains a grain and a half of arsenic, enough to kill a child.

4. Never put a pin in the mouth or between the teeth, for a single instant, because a sudden effort to laugh or speak, may convey it into the throat, or lungs, or stomach, causing death in a few minutes, or requiring the windpipe to be cut open to get it out; if it has passed into the stomach, it may, as it has done, cause years of suffering, ceasing only when it has made its way out of the body through the walls of the abdomen or other portion of the system.

5. It is best to have no button or string about any garment worn during the night. A long, loose night-gown is the best thing to sleep in. Many a man has facilitated an attack of apoplexy by buttoning his shirt-collar.

6. If you wake up of a cold night, and find yourself very restless, get out of bed, and standing on a piece of carpet or cloth of any kind, spend five or ten minutes in rubbing the whole body vigorously and rapidly with the hands, having previously thrown the bed clothing towards the foot of the bed so as to air both bed and body.

7 If you find that you have inadvertently eaten too much, instead of taking something to settle the stomach, thus adding to the load under which it already labors, take a continuous walk with just enough activity to keep up a very slight moisture or perspiration on the skin, and do not stop until entirely relieved, but end your exercise in a warm room, so as to cool off very slowly.

8. Never put on a pair of new boots or shoes on a journey, especially on a visit to the city; rather wear your easiest, oldest pair, otherwise you will soon be painfully disabled.

9. A loosely-fitting boot or shoe, while traveling in winter, will keep the feet warmer, without any stockings at all, than a tight pair, over the thickest, warmest hose.

10. Riding against a cold wind, immediately after singing or speaking in public, is suicide.

11. Many public speakers have been disabled for life by speaking under a hoarseness of voice.

12. If you happen to get wet in cold weather, keep moving on foot with a rapidity sufficient to keep off a feeling of chilliness until you get into a house, and not waiting to undress, drink instantly and plentifully of hot tea of some sort; then undress, wipe dry quickly, and put on warm, dry clothing.

13. Never go to bed with cold feet, if you want to sleep well.

14. If a person faints, place him instantly flat on a bed, or floor, or earth, on his back, and quietly let him alone at least for ten minutes; if it is simply a fainting-fit, the blood, flowing on a level will more speedily equalize itself throughout the system; cold water dashed in the face, or a sitting position are unnecessary and pernicious.

15. Never blow your nose, nor spit the product of a cough, nor throw a fruit peel on the sidewalk.

